# Probiotic-Mediated Biosynthesis of Silver Nanoparticles and Their Antibacterial Applications against Pathogenic Strains of *Escherichia coli* O157:H7

**DOI:** 10.3390/polym14091834

**Published:** 2022-04-29

**Authors:** Xiaoqing Wang, Sun-Young Lee, Shahina Akter, Md. Amdadul Huq

**Affiliations:** 1 Department of Food and Nutrition, College of Biotechnology and Natural Resource, Chung-Ang University, Anseong 17546, Gyeonggi-do, Korea; wxq2016@naver.com (X.W.); nina6026@cau.ac.kr (S.-Y.L.); 2 Department of Food Science and Biotechnology, Gachon University, Seongnam 461701, Gyeonggi-do, Korea; shahinabristy16@gmail.com

**Keywords:** probiotic-mediated biosynthesis, AgNPs, antibacterial applications, *E. coli* O157:H7

## Abstract

The present study aimed to suggest a simple and environmentally friendly biosynthesis method of silver nanoparticles (AgNPs) using the strain *Bacillus sonorensis* MAHUQ-74 isolated from kimchi. Antibacterial activity and mechanisms of AgNPs against antibiotic-resistant pathogenic strains of *Escherichia coli* O157:H7 were investigated. The strain MAHUQ-74 had 99.93% relatedness to the *B. sonorensis* NBRC 101234^T^ strain. The biosynthesized AgNPs had a strong surface plasmon resonance (SPR) peak at 430 nm. The transmission electron microscope (TEM) image shows the spherical shape and size of the synthesized AgNPs is 13 to 50 nm. XRD analysis and SAED pattern revealed the crystal structure of biosynthesized AgNPs. Fourier transform infrared spectroscopy (FTIR) data showed various functional groups associated with the reduction of silver ions to AgNPs. The resultant AgNPs showed strong antibacterial activity against nine *E. coli* O157:H7 pathogens. Minimum inhibitory concentration (MIC) values of the AgNPs synthesized by strain MAHUQ-74 were 3.12 μg/mL for eight *E. coli* O157:H7 strains and 12.5 μg/mL for strain *E. coli* ATCC 25922. Minimum bactericidal concentrations (MBCs) were 25 μg/mL for *E. coli* O157:H7 ATCC 35150, *E. coli* O157:H7 ATCC 43895, *E. coli* O157:H7 ATCC 43890, *E. coli* O157:H7 ATCC 43889, and *E. coli* ATCC 25922; and 50 μg/mL for *E. coli* O157:H7 2257, *E. coli* O157: NM 3204-92, *E. coli* O157:H7 8624 and *E. coli* O157:H7 ATCC 43894. FE-SEM analysis demonstrated that the probiotic-mediated synthesized AgNPs produced structural and morphological changes and destroyed the membrane integrity of pathogenic *E. coli* O157:H7. Therefore, AgNPs synthesized by strain MAHUQ-74 may be potential antibacterial agents for the control of antibiotic-resistant pathogenic strains of *E. coli* O157:H7.

## 1. Introduction

Silver nanoparticles (AgNPs) refer to nanoparticles with sizes ranging from 1 nm to 100 nm. AgNPs have been widely used in various applications in biomedical science such as in antibiotics, biosensors, drug delivery systems, antimicrobial, anticancer, and anti-inflammatory agents, etc. [1,2,3,4,5,6,7]. Many devices, including textiles, keyboards, and medical devices, now contain AgNPs that continuously release small amounts of silver ions to provide antibacterial protection [8]. AgNPs also have been implemented in the development of various bioactive materials, including polymer composites, because of their high antimicrobial activity. The polymer-based nanoparticles are usually a combination of organic polymers or a specific biomolecule in which an inorganic nanoparticle is embedded. Polymer-based metallic nanoparticles are widely explored due to their versatility, biodegradability, being environmentally friendly, and being biocompatible in nature [9,10]. The successful capping of polymers on AgNPs may prevent the agglomeration of synthesized nanocomposite as well as facilitate the stabilization process [11]. It was reported that AgNPs-containing polymer composite exhibits significant antimicrobial activity against pathogenic bacteria [12]. AgNPs have been used extensively in recent times to create antibacterial coatings [13,14,15]. Depending on the application, various shapes of nanoparticles can be constructed. Spherically shaped AgNPs are commonly used, but diamond, octagonal, and flake shapes are also used [16,17]. For synthesizing nanoparticles, various methods have been utilized, including physical, chemical, and biological methods [18]. The most commonly used physical approaches for nanoparticle synthesis are laser ablation and evaporation–condensation [19]. The advantages of these approaches are controllable chemical purity and the generation of clusters with free surfaces; in addition, the free choice of size has proven to be feasible [20]. However, a tubular-reactor-mediated physical synthesis at atmospheric pressure has some drawbacks. For example, a tube furnace requires a long time, large space, and enormous energy [19]. The chemical method provides a simple method to synthesize AgNPs in solution using organic and inorganic reducing agents. Typically, the chemical synthesis of AgNPs solution generally consists of the following three main components: (1) a metal precursor, (2) a reducing agent, and (3) a stabilizing/blocking agent. Silver nitrate is often used as the metal precursor, and sodium citrate and ascorbate, sodium borohydride (NaBH_4_), polyol process, elemental hydrogen, hydrazine, N, N-dimethylformamide (DMF), ascorbic acid, poly (ethylene glycol)-block copolymers, and ammonium formate are used as different reducing agents [21,22,23]. However, clinical and food applications avoid the use of chemical processes to produce nanoparticles, because these toxic chemicals are used in the process. In contrast, biosynthesis has many advantages, such as high stability, low toxicity to healthy cells, and no synthesis of toxic by-products [14]. Therefore, the use of biological pathways to synthesize nanoparticles is the preferred method, which eliminates the use of toxic chemicals, and is cost-effective and environmentally friendly [24].

Regarding the biosynthesis of AgNPs, the most commonly used materials are bacteria, fungi, algae, plants, and their components, etc. [14]. Two methods can be used for the biosynthesis of silver nanoparticles using microorganisms. According to the location of the synthesis of nanoparticles, they can be classified as intracellular or extracellular. Contrary to an intracellular synthesis that requires complicated purification steps, the extracellular synthesis of nanoparticles is more convenient, easy, and cost-effective. Therefore, extra-cellular synthesis is preferred because the removal of bacterial cells in advance simplifies the recovery of nanoparticles and can be easy and rapid purification of synthesized nanoparticles. Various bacteria including *Bacillus* sp., *Escherichia coli*, *Lactobacillus kimchicus*, *Pseudomonas deceptionensis, Solibacillus isronensis, Cedecea* sp., *Novosphingobium* sp., and *Sporosarcina koreensis* strains can reduce Ag+ ions to Ag and form AgNPs [25,26,27,28,29,30,31,32]. Biosynthesized AgNPs show superior properties such as hydrophilicity, stability, and large surface area. Compared with chemical synthesis routes, the bacterial-based synthesis method is economical, simple, reproducible, and requires less energy.

Fermented food is a type of food manufactured by humans ingeniously using beneficial microorganisms. Therefore, there are abundant microorganisms in fermented food, which provides a good source of microbial diversity. Various biomolecules are produced by bacteria including biopolymers such as polysaccharides, proteins, enzymes, and DNA, where the monomer units are sugars, amino acids, and nucleotides, respectively. These extracellular polymeric substances may play important roles in the synthesis and stabilization of nanoparticles and may enhance the efficacy of NPs [33]. Bacteria secrete various enzymes in the culture supernatant, including nitrate reductase responsible for nanoparticle biosynthesis. Different *Bacillus* sp. can secrete NADH- and NADH-dependent enzymes that involve in the reduction of silver ions in the form of AgNPs [25]. Members of the genus *Bacillus* have significant microbiological uses [34]. Numerous enzymes, antibiotics, and other metabolites have medical, agricultural, pharmaceutical, and other industrial applications [35]. For the biosynthesis of AgNPs, plant products such as fruit extract and soil bacteria are commonly used. There are a few studies to synthesize AgNPs using microorganisms isolated from foods. At present, drug-resistant pathogenic bacteria are a serious threat to humans. *Escherichia coli* O157:H7 is a notorious pathogen, and this pathogen is associated with a broad spectrum of illnesses in humans ranging from mild diarrhea and hemorrhagic colitis to the potentially fatal hemolytic uremic syndrome [36]. In this study, we report a newly isolated *B. sonorensis* MAHUQ-74 from kimchi, which we used for the synthesis of AgNPs using an extracellular method. The culture supernatant of *B. sonorensis* MAHUQ-74 was used to quickly and easily synthesize AgNPs without adding any reducing agent. Then, resultant AgNPs were characterized, and their antimicrobial activity at the mechanism level was investigated against various pathogenic strains of *E. coli* O157:H7.

## 2. Materials and Methods

### 2.1. Materials and Bacterial Strains

The culture medium was purchased from Difco, MB Cell (Seoul, South Korea). Analytical grade AgNO_3_ (silver nitrate) was purchased from Sigma-Aldrich Chemicals (St. Louis, MO, USA). Nine pathogenic *E. coli* O157:H7 strains were used in this study—namely, *E. coli* O157:H7 ATCC 43895, ATCC 35150, ATCC 43889, ATCC 43890, ATCC 43894, ATCC 25922; *E. coli* O157:H7 8624, 2257; and *E. coli* O157:NM 3204-92—which were collected from the bacterial culture collection of Chung-Ang University (Anseong-si, Korea). All strains were maintained at −80 °C in tryptic soy broth (TSB) containing 20% glycerol (*v*/*v*) and were activated by cultivation on LB agar for 24 h at 37 °C.

### 2.2. Isolation, Identification, and Characterization of AgNP-Producing Probiotics

Kimchi, a traditional, fermented food product, was collected from the retail market in Anseong, Korea. Briefly, 1 g of kimchi was dissolved in 9 mL of 0.85% (*w*/*v*) NaCl solution, serially diluted up to 10^−5^, and 100 µL of each dilution was spread on both nutrient agar (NA) and Reasoner’s 2A agar (R_2_A agar) plates [37]. Then, the plates were incubated at 37 °C for 3 days. Single colonies were purified by successive transferring to new NA or R_2_A agar plates. AgNP synthesis ability was screened by culturing all isolates in 5 mL R2A broth media for 48 h at 37 °C. Then, 1 mM AgNO_3_ solution was added to the culture supernatant and incubated again for 48 h. Among all of these isolated strains, strain MAHUQ-74 showed strong AgNP synthesis ability. Then, the strain was identified by 16S rRNA gene sequence analysis [38]. The 16S rRNA gene sequence of strain MAHUQ-74 was submitted to GeneBank of NCBI. The 16S rRNA gene sequences of related taxa were obtained from the EzBioCloud server [39]. The phylogenetic tree was created using the MEGA6 program [40] and the neighbor-joining method, to discover the phylogenetic location of isolated strain MAHUQ-74 [41]. The optimum growth conditions of strain MAHUQ-74 including media, temperature and pH, gram-straining reaction, catalase, and oxidase activities were examined according to the previous description [42]. According to the manufacturer’s instructions, commercial API ZYM and API 20 NE kits (bioMérieux) were used to further determine enzyme activity and carbon source utilization. The probiotic strain MAHUQ-74 was deposited to the Korean Agricultural Culture Collection (KACC).

### 2.3. Biosynthesis of AgNPs Using Probiotic Strain MAHUQ-74

For the biosynthesis of AgNPs, the *B. sonorensis* MAHUQ-74 strain was cultured in 100 mL of R_2_A broth and incubated at 37 °C for 3 days in a shaking incubator at 180 rpm. After three days of incubation, the culture supernatant was collected by centrifugation at 10,000 rpm, 4 °C for 10 min in a centrifuge. Then, 0.1 mL (1 M concentration) filter-sterilized AgNO_3_ solution was added to 100 mL supernatant and incubated again in an orbital shaker at 180 rpm and 30 °C for 24 h. The synthesis of AgNPs was confirmed by visual observation of the color change. Finally, the biosynthesized AgNPs were collected by centrifugation at 13,000 for 30 min. The precipitate of synthesized AgNPs was washed with distilled water. Then, the air-dried samples were used for characterization and antibacterial studies.

### 2.4. Characterization of Biosynthesized AgNPs

The biosynthesis of AgNPs was monitored by a UV–Vis spectrophotometer in the range of 300–800 nm. By using field-emission transmission electron microscopy (FE-TEM), energy-dispersive X-ray (EDX) spectroscopy, element map, and selective area diffraction (SAED) mode, the morphology, element composition, and purity of the synthesized AgNPs were studied. A drop of AgNP solution was kept on a copper mesh, dried at room temperature, and finally transferred to a microscope for analysis. X-ray diffraction (XRD) analysis was conducted with an X-ray diffractometer (D8 Advance, Bruker, Germany) in the range of 30–90° over 2θ value, using CuKα radiation, at 40 kV, 40 mA with 6°/min scanning rate. For XRD analysis, air-dried AgNP samples were used. Fourier transform-infrared (FTIR) spectroscopy showed biomolecules related to the biosynthesis and stability of AgNPs. FTIR analysis was performed by using a PerkinElmer Fourier Transform Infrared Spectrometer (PerkinElmer Inc., Waltham, MA, USA), with a resolution of 4 cm^–1^ and a range of 400–4000 cm^–1^. A Malvern Zetasizer Nano ZS90 (Otsuka Electronics, Osaka, Japan) was used to determine the particle size of the green synthetic AgNPs by dynamic light scattering (DLS) at 25 °C. Pure water was used as a dispersion medium with a dielectric constant of 78.3, a viscosity of 0.8878 cP, and a refractive index of 1.3328.

### 2.5. Antimicrobial Activity of Probiotic-Mediated Synthesized AgNPs

The tested pathogenic microbes (nine different *E. coli* O157:H7 strains) were grown overnight in LB broth. Briefly, 100 µL of the bacterial culture sample of the tested pathogen was spread on the LB agar plate. Sterile paper discs containing AgNPs 50 µL and 100 µL (1000 μg/mL) were placed on the LB agar plates. Then, the plates were incubated in an incubator at 37 °C for 24 h. Similarly, the antibacterial activity of some commercial antibiotics such as erythromycin (15 μg/disc), vancomycin (30 μg/disc), and penicillin G (10 μg/disc) was tested against nine *E. coli* O157:H7 strains. The inhibition zones were calculated after 24 h of incubation. The test was performed twice.

### 2.6. Determination of MIC and MBC

The minimum inhibitory concentration (MIC) of probiotic-mediated synthesized AgNPs was measured using the broth microdilution technique. Nine *E. coli* O157:H7 strains were grown in LB broth overnight at 37 °C, and the turbidity was fixed at around 1 × 10^6^ CFUs/mL. Then, 100 mL of test bacterial (1 × 10^6^ CFUs/mL) suspension were added to a 96-well plate, after which an equal volume of AgNP solution with various concentrations (3.12–200 μg/mL) was added, and finally, the plates were incubated in a 37 °C incubator for 24 h. Every 3 h of the interval, the absorbance (at 600 nm) was measured in a microplate reader. Minimum bactericidal concentration (MBC) was determined by streaking 8 µL of each set on an agar plate and again incubated for 24 h at 37 °C. The culture plates were observed by direct visualization to determine the MBC that blocked bacterial growth [24].

### 2.7. Morphological Evaluation of Treated Cells Using FE-SEM

The morphological alterations of *E. coli* O157:H7 (ATCC 35150) were examined using FE-SEM. Logarithmic growth phase cells (1 × 10^7^ CFU/mL) were treated with probiotic-mediated synthesized AgNPs at a concentration of 1 × MBC for 6 h. In the control, the bacterial culture was treated without AgNP solution. The treated cells were washed with phosphate-buffered saline (PBS). The cells were fixed for 4 h using 2.5% glutaraldehyde solution and then, washed several times with PBS. Again, with 1% tetroxide cells were fixed and washed with PBS buffer solution. The fixed cells were dehydrated using different concentrations of ethanol (30 to 100%, every 10% interval) at room temperature for 10 min. Then, the samples were dried with a dryer. Finally, the samples were placed on the SEM metal grid and coated with gold. The morphological and structural changes in the cells were observed using FE-SEM (S-4700, Hitachi, Tokyo, Japan).

## 3. Results and Discussion

### 3.1. Isolation, Identification, and Characterization of AgNP-Producing Probiotic

**Figure 1:**
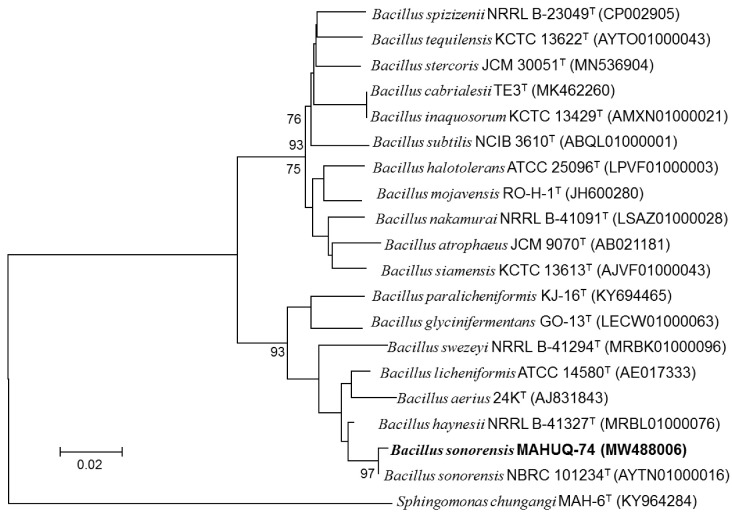
The neighbor-joining (NJ) tree is based on 16S rRNA gene sequence analysis, showing the position of *Bacillus sonorensis* MAHUQ-74 and other *Bacillus* species.

Strain MAHUQ-74 was isolated from kimchi, a traditional, fermented food product. Strain MAHUQ-74 was deposited to the Korean Agriculture Culture Collection Center (Deposition number: KACC 22255). The 16S rRNA gene sequence of strain MAHUQ-74 was 1471 bp and the sequence was deposited to NCBI (Accession number: MW488006). Based on the 16S rRNA gene sequence analysis, strain MAHUQ-74 showed the highest sequence similarity with *B. sonorensis* NBRC 101234T (99.93%). To find the phylogenetic location of strain MAHUQ-74, the phylogenetic tree was constructed using the neighbor-joining method in the MEGA6 program package (Figure 1). Cells were Gram-positive, catalase-positive, and oxidase-negative, and were able to grow within 30–50 °C (optimum 37 °C). The strain was positive for hydrolysis of casein, gelatin, starch, and esculin. In the API 20 NE strip, nitrate could be reduced to nitrite; D-glucose was fermented; indole was not produced; urease, arginine dihydrolase, and gelatinase activities were absent; α-glucosidase, α-galactosidase, and β-glucosidase activity were present; and D-glucose, N-acetyl-glucosamine, L-arabinose, D-maltose, D-mannitol, and D-mannose were assimilated. Capric acid, adipic acid, and phenylacetic acid were not assimilated. In the API ZYM strip, alkaline phosphatase, esterase (C4), alkaline phosphatase, leucine arylamidase, naphthol-AS-BI-phosphohydrolase, esterase lipase (C8), acid phosphatase, α-glucosidase, α-galactosidase, and β-glucosidase were present. Lipase (C14), valine arylamidase, cystine arylamidase, trypsin, α-chymotrypsin, β-galactosidase, N-acetyl-β-glycosaminidase, α-mannosidase, and α-fucosidase were absent (Table 1).

### 3.2. Biosynthesis of AgNPs Using Strain MAHUQ-74

**Figure 2:**
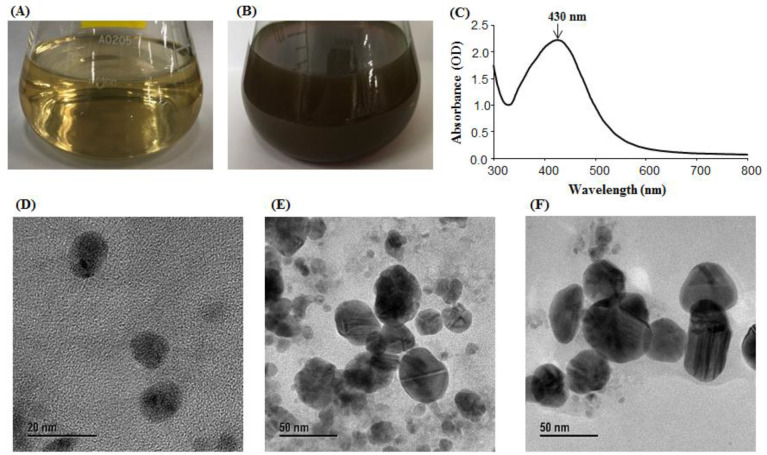
R2A broth as control (**A**), synthesized AgNPs (**B**), UV–Vis spectra (**C**), and FE-TEM images of synthesized AgNPs (**D**–**F**).

Biosynthesis of AgNPs using *B. sonorensis* MAHUQ-74 was ensured by monitoring the color of the culture supernatant. The color of the MAHUQ-74 culture supernatant turned to deep brown from watery yellow, which indicated the synthesis of AgNPs. The control sample (without bacterial supernatant) did not show any color change when incubated under the same conditions (Figure 2A,B). Optimum temperature (30 °C) and AgNO_3_ concentration (final concentration 1 mM) for stable synthesis were determined based on the ultraviolet–visible spectroscopy (UV–Vis) analysis. Two methods are commonly utilized for the biosynthesis of nanoparticles using bacteria—intracellular and extracellular methods. The intracellular method is a more complex and a multi-step process, compared with the extracellular method. In the current study, the extracellular methodology was used to biosynthesize AgNPs, using probiotic bacterial strain MAHUQ-74, which was simple, facile, cost-effective, and ecofriendly.

### 3.3. Characterization of Biosynthesized AgNPs

Probiotic-mediated synthesized AgNPs showed a peak at 430 nm (Figure 2C), which revealed that AgNPs were fruitfully synthesized. AgNPs are known to exhibit a UV–Visible absorption peak in the range of 400–500 nm [24]. The lower absorption wavelength indicates smaller-sized, spherical NPs [43], which suggested that MAHUQ-74 might synthesize small-sized AgNPs. The results indicated that MAHUQ-74 may be a promising candidate for the biosynthesis of AgNPs. TEM analysis showed that AgNPs were spherical and elliptical, and they dispersed well, without obvious agglomeration. The size of the synthesized AgNPs was 13–50 nm (Figure 2D–F).

**Figure 3:**
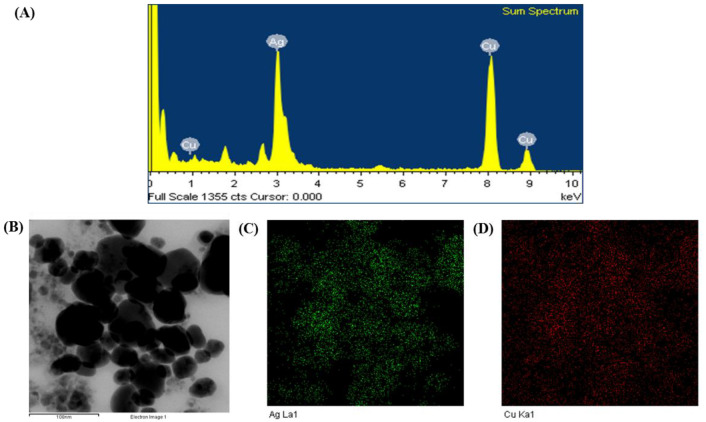
Energy dispersive X-ray (EDX) spectrum of synthesized AgNPs (**A**), FE-TEM image used for elemental mapping (**B**), distribution of silver (**C**), and copper (**D**) in elemental mapping.

The composition and purity of biosynthesized AgNPs were investigated using an EDX spectrometer. The EDX data revealed elemental signals of silver atoms in probiotic-mediated synthesized AgNPs at around 3 keV and indicated the homogenous distribution of AgNPs. Some other peaks of copper were also found in the EDX mode due to the use of a copper grid (Figure 3A). The elemental mapping results showed that the most distributed element in biosynthetic nanoproducts was silver (Figure 3B–D, Table 2).

**Figure 4:**
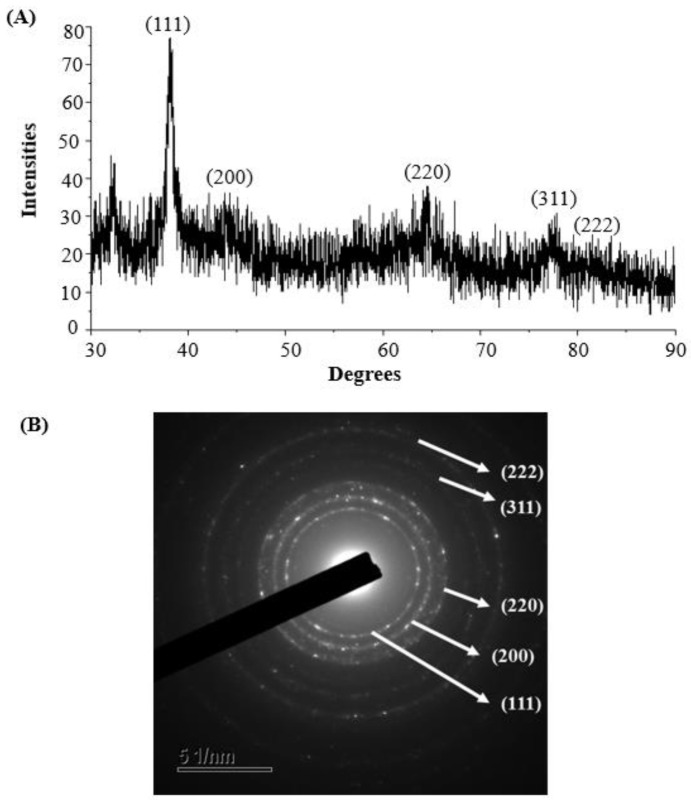
XRD pattern (**A**) and SAED pattern (**B**) of probiotic-mediated synthesized AgNPs.

The XRD data showed diffraction peaks at 2θ values of 38.163°, 44.247°, 64.519°, 77.804°, and 81.474°, which matched with the lattice planes of AgNPs (111, 200, 220, 311, and 222, respectively) (Figure 4A). A recently reported study revealed similar XRD results, in which AgNPs were synthesized using microorganisms [44]. The crystalline structure of probiotic-mediated synthesized AgNPs was confirmed using SAED analysis, which revealed sharp rings corresponding to the reflections of 111, 200, 220, 311, and 222 (Figure 4B). Both the XRD spectrum and SAED pattern confirmed the crystalline structure of AgNPs.

**Figure 5:**
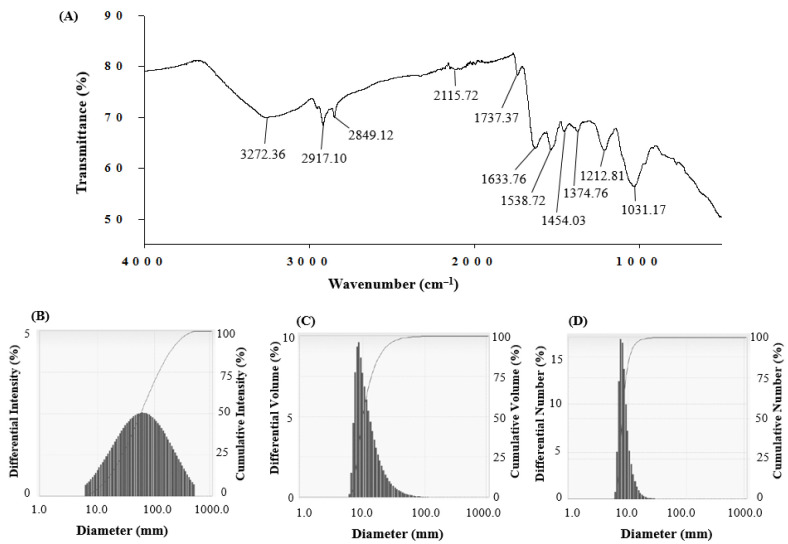
FTIR spectra (**A**) and particle size distribution according to intensity (**B**), volume (**C**), and number (**D**) of synthesized AgNPs.

FTIR analysis revealed that different functional groups surrounded the biosynthesized AgNPs (Figure 5A). The FTIR pattern of AgNPs had several peaks at 3272.36 cm^–1^, 2917.11 cm^–1^, 2849.12 cm^–1^, 2115.72 cm^–1^, 1737.37 cm^–1^, 1633.76 cm^–1^, 1538.72 cm^–1^, 1454.03 cm^–1^, 1374.76 cm^–1^, 1212.81 cm^–1^, and 1031.17 cm^–1^. The band found at 3272.36 cm^–1^ was attributed to O–H (alcohol) and/or N–H (amine) stretching. The bands at 2917.11 and 2849.12 cm^–1^ were attributed to C–H (alkane) group. The peaks observed at 2115.72 cm^–1^ were attributed to the N=C=S (isothiocyanate) stretching. The peak found at 1737.37 cm^–1^ represented the C=O (α, β-unsaturated ester) stretching. The peaks at 1633.76, 1538.72, and 1454.03 cm^−1^ were attributed to C=C (olefin) stretching, N-O (nitro compound) stretching, and methyl group C–H (alkane) bending, respectively. The peak observed at 1374.76 cm^–1^ represented the O-H (phenol) bending. The bands at 1212.81 cm^–1^ and 1031.17 cm^–1^ were attributed to C-O (alkyl aryl ether) stretching and S=O (sulfoxide) stretching, respectively. The FTIR spectrum indicated that the functional molecules including biopolymers such as proteins, enzymes, and amino acids secreted by probiotic bacteria could be involved in both the synthesis and stabilization of AgNPs. The particle size of biosynthesized AgNPs was measured with dynamic light scattering (DLS) analysis. Figure 5B shows the particle size distribution of biosynthesized AgNPs based on intensity, volume, and number. The average particle size of probiotic-mediated synthesized AgNPs was 44.6 nm, and the polydispersity value was 0.406.

### 3.4. Antimicrobial Activity of Probiotic-Mediated Synthesized AgNPs against Different E. coli O157:H7 Strains

The antimicrobial activity of AgNPs is related to their ability to bind microbial DNA, proteins, and enzymes, as well as to alter cell morphology and function [18,45,46]. Small AgNPs have higher antimicrobial activity than large particles, allowing the faster release of Ag+ ions [47]. Many reports have tested the antimicrobial activity of biosynthetic AgNPs against different pathogenic microorganisms, including bacteria, fungi, yeasts, and microbial biofilms. In this study, AgNPs were synthesized using probiotic bacteria *B. sonorensis* MAHUQ-74, and their antibacterial ability was investigated against nine foodborne pathogenic *E. coli* O157:H7 strains (Table 3).

**Figure 6:**
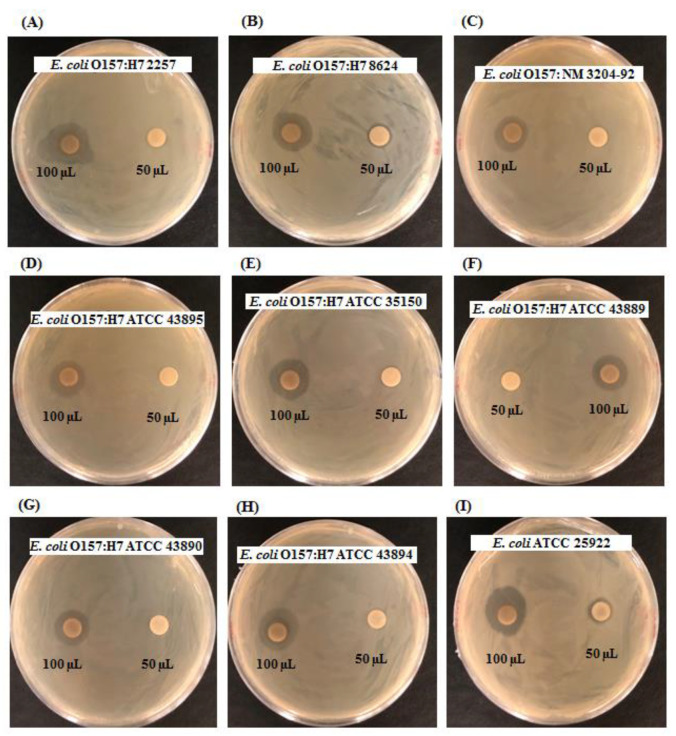
Antibacterial activity of synthesized AgNPs at 1000 μg/mL concentrations in the water against *E. coli* O157:H7 strains (**A**–**I**).

The results showed that the synthesized AgNPs had significant antibacterial activity against all tested foodborne pathogens. The antibacterial efficacy against various pathogenic strains of *E. coli* O157:H7 was determined by calculating the diameter of the zone of inhibition (Figure 6, Table 4). It was also found that AgNPs can inhibit the growth of most *E. coli* O157:H7 strains when treated with a 100 µL AgNP solution. The results of this study revealed that the probiotic bacteria *B. sonorensis* MAHUQ-74-mediated biosynthesized AgNPs are able to control foodborne pathogenic bacteria, especially pathogenic *E. coli* O157:H7 strains.

**Figure 7:**
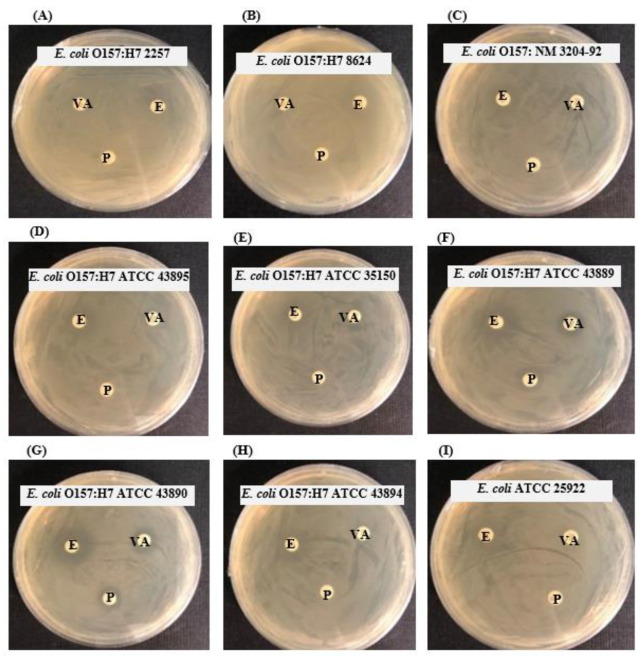
Antibacterial activity of some commercial antibiotics against nine *E. coli* O157:H7 strains (**A**–**I**). Abbreviations: P (penicillin G, 10 μg/disc), E (erythromycin, 15 μg/disc), and VA (vancomycin, 30 μg/disc).

Three antibiotics (erythromycin, vancomycin, and penicillin G) were also tested against the foodborne pathogens *E. coli* O157:H7. Six of the nine foodborne pathogens were found to be resistant to all three antibiotics included in this study (Figure 7, Table 5).

### 3.5. Determination of MIC and MBC

**Figure 8:**
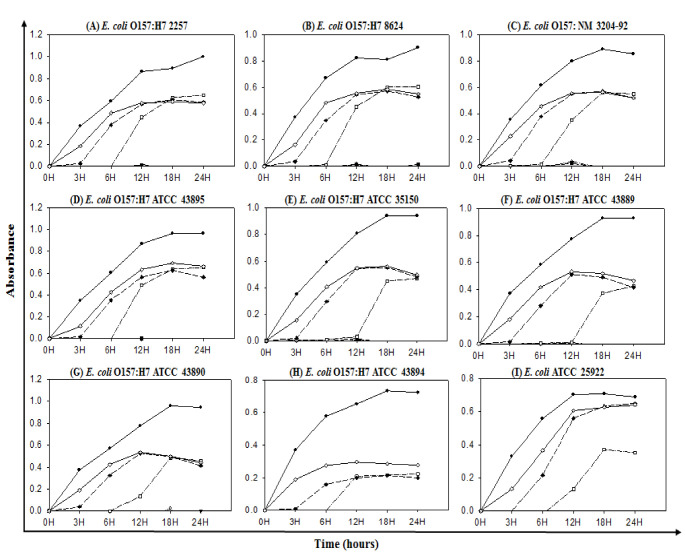
Growth curves of nine *E. coli* O157:H7 strains (**A**–**I**) cultured in LB broth with various concentrations of synthesized AgNPs to determine MIC. Control (●); 200 μg/mL (◦); 100 μg/mL (▼) 50 μg/mL (△); 25 μg/mL (§); 12.5 μg/mL (□); 6.25 μg/mL (w); 3.12 μg/mL (◊).

The MIC and MBC of probiotic-mediated synthesized AgNPs against a total of nine *E. coli* O157:H7 were determined by a standard broth dilution assay. Bacterial growth curves revealed that the MICs of biosynthesized AgNPs were 3.12 μg/mL for eight *E. coli* O157:H7 strains (Figure 8A–H) and 12.5 μg/mL for strain *E. coli* ATCC 25,922 (Figure 8I). This result indicated that the probiotic-mediated synthesized AgNPs extremely suppressed the growth of pathogenic strains of *E. coli* O157:H7.

**Figure 9:**
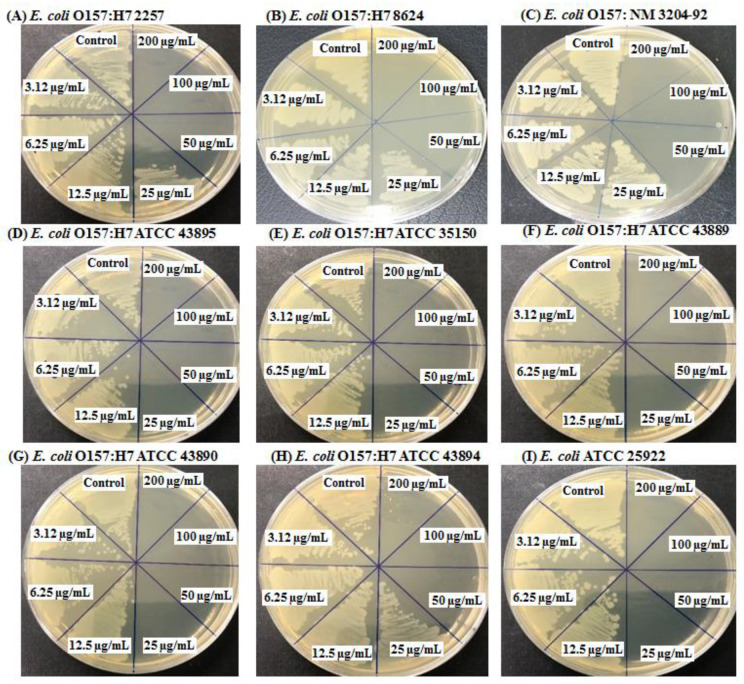
MBC of probiotic-mediated synthesized AgNPs against *E. coli* O157:H7 strains (**A**–**I**).

The MBC of synthesized AgNPs was 25 μg/mL for *E. coli* O157:H7 ATCC 43895, *E. coli* O157:H7 ATCC 35150, *E. coli* O157:H7 ATCC 43889, *E. coli* O157:H7 ATCC 43,890, and *E. coli* ATCC 25,922, and 50 μg/mL for *E. coli* O157:H7 2257, *E. coli* O157: NM 3204-92, *E. coli* O157:H7 8624 and *E. coli* O157:H7 ATCC 43,894 (Figure 9A–I).

### 3.6. Study of Morphogenesis of E. coli O157:H7-Treated Cells Using FE-SEM

**Figure 10:**
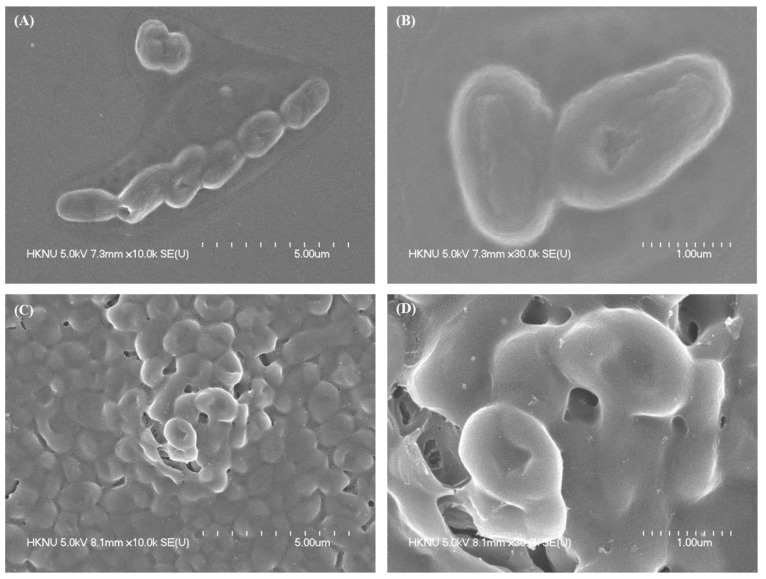
FE-SEM images of normal *E. coli* O157:H7 (ATCC 35150) (**A**,**B**) and 1 × MBC probiotic-mediated synthesized AgNPs treated *E. coli* O157:H7 (ATCC 35150) (**C**,**D**).

The structural and morphological alterations of *E. coli* O157:H7 ATCC 35,150 cells treated with probiotic-mediated synthesized AgNPs were directly observed using FE-SEM (Figure 10). Based on the FE-SEM analysis, it was found that the untreated *E. coli* O157:H7 cells were intact, normal, and rod-shaped, and the structural integrity of the bacterial cells was good without any damage (Figure 10A,B). However, after treatment with 1 × MBC of synthesized AgNPs, the shape of *E. coli* O157:H7 cells became abnormal, irregular, and wrinkled, with the cell membrane entirely collapsed and damaged (Figure 10C,D).

Although the bactericidal mechanism of AgNPs is not yet fully understood, several hypotheses have been proposed in the literature. The attachment of AgNPs to the bacterial cell membrane results in the formation of a “pit” on the bacterial cell wall, thereby allowing the nanoparticles to enter the bacterial cells in the periplasm [48,49]. As a result, subsequent changes in the DNA of the bacterial cells treated with AgNPs lead to the loss of DNA replication ability, which leads to the inactivation of the expression of proteins and enzymes necessary for ATP production. In the present study, the structural and morphological alterations, damage to bacterial cell walls, and cell membrane indicated that the biosynthesized AgNPs might interfere with the metabolic process and normal cell functions, leading to the death of bacterial cells.

## 4. Conclusions

In conclusion, the biological compounds produced by bacteria have high utility value and may play important roles in the production of AgNPs. Biomolecules, including biopolymers such as proteins, enzymes, and amino acids, that are secreted by bacteria are involved in the synthesis process but also may improve the function of synthesized AgNPs. In the present study, we isolated and identified the strain *B. sonorensis* MAHUQ-74 and used their culture supernatant for the biosynthesis of AgNPs. The AgNPs were analyzed using UV–Vis, FE-TEM, XRD, EDX, FTIR, and DLS. FE-TEM images indicated that the AgNPs were mostly circular, and the size ranged from 13 to 50 nm. The FTIR data indicated that various biomolecules may participate in the synthesis and stabilization of AgNPs. The extracellular method was used for the biosynthesis of AgNPs. Moreover, the biosynthesized AgNPs showed potent antibacterial efficacy against nine pathogenic *E. coli* O157:H7 strains. The MICs of the AgNPs synthesized by strain MAHUQ-74 were 3.12 to 12.5 μg/mL for the tested nine *E. coli* O157:H7 strains. The MBCs of the AgNPs synthesized by strain MAHUQ-74 were 25 to 50 μg/mL for the tested *E. coli* O157:H7 strains. FE-SEM analysis showed that the biosynthesized AgNPs can cause changes in the morphology and structure of the foodborne pathogenic *E. coli* O157:H7 strain and destroy the integrity of the membrane, leading to cell death. This is the first study on the use of probiotic *B. sonorensis* MAHUQ-74 isolated from kimchi for the rapid and facile synthesis of bioactive AgNPs. AgNPs manufactured using *B. sonorensis* MAHUQ-74 may be potential antimicrobial agents for controlling antibiotic-resistant microorganisms, especially pathogenic strains of *E. coli* O157:H7.

## Figures and Tables

**Table 1:** Biochemical characterization of *Bacillus sonorensis* MAHUQ-74.

API 20 NE	Result	API ZYM	Result
Reduction of nitrate (API 20 NE)	+	Esterase (C4)	w
		Esterase lipase (C8)	w
		Alkaline phosphatase	+
		Lipase (C-14)	-
Hydrolysis of:		Acid phosphatase	w
Esculine	+	Leucine arylamidase	+
Gelatine	+	Valine arylamidase	-
Urea	-	Cystine arylamidase	-
4-nitrophenyl-BD-galactopyranoside	+	Trypsin	-
Utilization of:		α-chymotrypsin	-
D-glucose	+	β-glucuronidase	-
D-maltose	+	Naphthol-AS-BI-phosphohydrolase	w
D-mannose	+	N-acetyl-β-glucosaminidase	-
D-mannitol	+	α-mannosidase	-
L-arabinose	+	α-glucosidase	+
N-acetyl-glucosamine	+	α-galactosidase	+
Trisodium citrate	+	β-glucosidase	+
Phenylacetic acid	-	β-galactosidase	-
		α-fucosidase	-
+, Positive; -, Negative; w, Weakly positive.			

**Table 2:** The number and percentage of chemical elements present in EDX spectrum of probiotic-mediated synthesized AgNPs.

Element	Weight %	Atomic %
Cu K	39.77	52.85
Ag L	60.23	47.15
Totals	100.00	100.00

**Table 3:** *E. coli* O175:H7 strains used for antibacterial experiments.

No.	Strains	Origin
1	*Escherichia coli* O157:H7 2257	*E. coli* O157:H7 FDIU strain
2	*Escherichia coli* O157:H7 8624	Wild-type *E. coli* O157:H7, a clinical isolate
3	*Escherichia coli* O157: NM 3204-92	International vaccine institute clinical specimen
4	*Escherichia coli* O157:H7 ATCC 43895	About the outbreak of raw hamburger and hemorrhagic colitis
5	*Escherichia coli* O157:H7 ATCC 35150	Feces, human
6	*Escherichia coli* O157:H7 ATCC 43889	Isolated from the stool of a patient with hemolytic uremic syndrome in North Carolina
7	*Escherichia coli* O157:H7 ATCC 43890	Human feces, California
8	*Escherichia coli* O157:H7 ATCC 43894	Human feces from an outbreak of hemorrhagic colitis, Michigan
9	*Escherichia coli* ATCC 25922	Clinical isolate
FDIU, Federal Disease Investigation Unit (Washington State University)		

**Table 4:** Antibacterial efficacy of probiotic-mediated synthesized AgNPs against *E. coli* O157:H7 strains.

No.	Pathogenic Strains	Zone of Inhibition(mm)	
		50 µL	100 µL
1	*E. coli* O157:H7 2257	10.1	24
2	*E. coli* O157:H7 8624	10.1	17
3	*E. coli* O157: NM 3204-92	10.1	16
4	*E. coli* O157:H7 ATCC 43895	-	16
5	*E. coli* O157:H7 ATCC 35150	10	18
6	*E. coli* O157:H7 ATCC 43889	10.1	16
7	*E. coli* O157:H7 ATCC 43890	10	16
8	*E. coli* O157:H7 ATCC 43894	10	19
9	*E. coli* ATCC 25922	10.1	22
-, no inhibition zone			

**Table 5:** Antibacterial efficacy of some commercial antibiotics against *E. coli* O157:H7 strains—no inhibition zone.

No.	Pathogenic Species	Antibiotic	Zone of Inhibition (mm)
1	*E. coli* O157:H7 2257	Erythromycin	-
		Vancomycin	-
		Penicillin G	-
2	*E. coli* O157:H7 8624	Erythromycin	-
		Vancomycin	-
		Penicillin G	-
3	*E. coli* O157: NM 3204-92	Erythromycin	-
		Vancomycin	-
		Penicillin G	-
4	*E. coli* O157:H7 ATCC 43895	Erythromycin	-
		Vancomycin	-
		Penicillin G	-
5	*E. coli* O157:H7 ATCC 35150	Erythromycin	-
		Vancomycin	-
		Penicillin G	-
6	*E. coli* O157:H7 ATCC 43889	Erythromycin	15
		Vancomycin	10
		Penicillin G	9
7	*E. coli* O157:H7 ATCC 43890	Erythromycin	17
		Vancomycin	10
		Penicillin G	9
8	*E. coli* O157:H7 ATCC 43894	Erythromycin	10
		Vancomycin	8
		Penicillin G	-
9	*E. coli* ATCC 25922	Erythromycin	-
		Vancomycin	-
		Penicillin G	-

## Data Availability

Not applicable.

## References

[1] Kedi P.B.E., Meva F.E., Kotsedi L. (2018). Eco-friendly synthesis, characterization, in vitro and in vivo anti-inflammatory activity of silver nanoparticle-mediated *Selaginella myosurus* aqueous extract. Int. J. Nanomed..

[2] El-Naggar N.E., Hussein M.H., El-Sawah A.A. (2017). Bio-fabrication of silver nanoparticles by phycocyanin, characterization, in vitro anticancer activity against breast cancer cell line and in vivo cytotoxicity. Sci. Rep..

[3] Fouda A., Abdel-Maksoud G., Abdel-Rahman M.A., Salem S.S., Hassan S.E.D., El-Sadany M.A.H. (2019). Eco-friendly approach utilizing green synthesized nanoparticles for paper conservation against microbes involved in biodeterioration of archaeological manuscript. Int. Biodeterior. Biodegrad..

[4] Fouda A., Abdel-Maksoud G., Abdel-Rahman M.A., Eid A.M., Barghoth M.G., El-Sadany M.A.H. (2019). Monitoring the effect of biosynthesized nanoparticles against biodeterioration of cellulose-based materials by *Aspergillus niger*. Cellulose.

[5] Cheon J.Y., Kim S.J., Rhee Y.H., Kwon O.H., Park W.H. (2019). Shape-dependent antimicrobial activities of silver nanoparticles. Int. J. Nanomed..

[6] Burduසel A.C., Gherasim O., Grumezescu A.M., Mogoanta L., Ficai A., Andronescu E. (2018). Biomedical applications of silver nanoparticles: An up-to-date overview. Nanomaterials.

[7] Siddiqi K.S., Husen A., Rao R.A.K. (2018). A review on biosynthesis of silver nanoparticles and their biocidal properties. J. Nanobiotechnology.

[8] Kandarp M., Mihir S. (2013). Synthesis of silver nanoparticles by using sodium borohydride as a reducing agent. Int. J. Eng. Res. Technol..

[9] Al-Jumaili A., Mulvey P., Kumar A. (2019). Eco-friendly nanocomposites derived from geranium oil and zinc oxide in one step approach. Sci. Rep..

[10] Wang L.S., Wang C.Y., Yang C.H., Hsieh C.L., Chen S.Y., Shen C.Y., Wang J.J., Huang K.S. (2015). Synthesis and anti-fungal effect of silver nanoparticles-chitosan composite particles. Int. J. Nanomed..

[11] Raza S., Ansari A., Siddiqui N.N., Ibrahim F., Abro M.I., Aman A. (2021). Biosynthesis of silver nanoparticles for the fabrication of non-cytotoxic and antibacterial metallic polymer based nanocomposite system. Sci. Rep..

[12] Quintero-Quiroz C., Botero L.E., Zárate-Triviño D., Acevedo-Yepes N., Escobar J.S., Pérez V.Z., Cruz-Riano L.J. (2020). Synthesis and characterization of a silver nanoparticle-containing polymer composite with antimicrobial abilities for application in prosthetic and orthotic devices. Biomater. Res..

[13] Huq M.A. (2020). Biogenic Silver Nanoparticles Synthesized by *Lysinibacillus xylanilyticus* MAHUQ-40 to control antibiotic-resistant human pathogens *Vibrio parahaemolyticus* and Salmonella Typhimurium. Front. Bioeng. Biotechnol..

[14] Poulose S., Panda T., Nair P.P., Théodore T. (2014). Biosynthesis of silver nanoparticles. J. Nanosci. Nanotechnol..

[15] Akter S., Lee S.Y., Siddiqi M.Z., Balusamy S.R., Ashrafudoulla M., Rupa E.J., Huq M.A. (2020). Ecofriendly synthesis of silver nanoparticles by *Terrabacter humi* sp. nov. and their antibacterial application against antibiotic-resistant pathogens. Int. J. Mol. Sci..

[16] Abou E.N., Kholoud M.M., Eftaiha A.A., Al-Warthan A., Ammar R.A. (2010). Synthesis and applications of silver nanoparticles. Arab. J. Chem..

[17] David D. (2008). The relationship between biomaterials and nanotechnology. Biomaterials.

[18] Huq M.A., Ashrafudoulla M., Rahman M.M., Balusamy S.R., Akter S. (2022). Green synthesis and potential antibacterial applications of bioactive silver nanoparticles: A review. Polymers.

[19] Iravani S., Korbekandi H., Mirmohammadi S.V., Zolfaghari B. (2014). Synthesis of silver nanoparticles: Chemical, physical and biological methods. Res. Pharm. Sci..

[20] Kruis F.E., Fissan H., Rellinghaus B. (2000). Sintering and evaporation characteristics of gas-phase synthesis of size-selected PbS nanoparticles. Mater. Sci. Eng. B.

[21] Tran Q.H., Le-Adv A.T. (2013). Silver nanoparticles: Synthesis, properties, toxicology, applications, and perspectives. Nat. Sci. Nanosci. Nanotechnol..

[22] Nasrollahzadeh M. (2014). Green synthesis and catalytic properties of palladium nanoparticles for the direct reductive amination of aldehydes and hydrogenation of unsaturated ketones. New J. Chem..

[23] Firdhouse M.J., Lalitha P.J. (2015). Biosynthesis of silver nanoparticles and its applications. J. Nanotechnol..

[24] Huq M.A. (2020). Green synthesis of silver nanoparticles using *Pseudoduganella eburnea* MAHUQ-39 and their antimicrobial mechanisms investigation against drug resistant human pathogens. Int. J. Mol. Sci..

[25] Yurtluk T., Akçay F.A., Avcı A. (2018). Biosynthesis of silver nanoparticles using novel *Bacillus* sp. SBT8. Prep. Biochem. Biotechnol..

[26] El-Dein M.M.N., Baka Z.A., Abou-Dobara M.I., El-Sayed A.K., El-Zahed M.M. (2021). Extracellular biosynthesis, optimization, characterization, and antimicrobial potential of *Escherichia coli* D8 silver nanoparticles. Biotech. Food Sci..

[27] Markus J., Mathiyalagan R., Kim Y.-J., Abbai R., Singh P., Ahn S., Perez Z.E.J., Hurh J., Yang D.C. (2016). Intracellular synthesis of gold nanoparticles with antioxidant activity by probiotic *Lactobacillus kimchicus* DCY51T isolated from Korean kimchi. Enzym. Microb. Technol..

[28] Jo J.H., Singh P., Kim Y.J., Wang C., Mathiyalagan R., Jin C.-G., Yang D.C. (2015). *Pseudomonas deceptionensis* DC5-mediated synthesis of extracellular silver nanoparticles. Artif. Cells Nanomed. Biotechnol..

[29] Singh P., Pandit S., Mokkapati S., Garnæs J., Mijakovic I. (2020). A Sustainable approach for the green synthesis of silver nanoparticles from *Solibacillus isronensis* sp. and their application in biofilm inhibition. Molecules.

[30] Singh P., Pandit S., Jers C., Abhayraj S., Garnæs J., Mijakovic I. (2021). Silver nanoparticles produced from *Cedecea sp*. exhibit antibiofilm activity and remarkable stability. Sci. Rep..

[31] Du J., Sing H., Yi T.H. (2017). Biosynthesis of silver nanoparticles by *Novosphingobium* sp. THG-C3 and their antimicrobial potential. Artif. Cells. Nanomed. Biotechnol..

[32] Singh P., Singh H., Kim Y.J. (2016). Extracellular synthesis of silver and gold nanoparticles by *Sporosarcina koreensis* DC4 and their biological applications. Enzym. Microb. Technol..

[33] Costa O., Raaijmakers J.M., Kuramae E.E. (2018). Microbial extracellular polymeric substances: Ecological function and impact on soil aggregation. Front. Microbiol..

[34] Turnbull P.C.B. (1996). Bacillus: Barron’s Medical Microbiology.

[35] Mandell G.L., Bennett J.E., Dolin R., Livingstone C. (2010). Mandell, Douglas, and Bennett’s. Principles and Practice of Infectious Diseases.

[36] Rahal E.A., Kazzi N., Nassar F.J., Matar G.M. (2012). *Escherichia coli* O157:H7-Clinical aspects and novel treatment approaches. Front. Cell Infect. Microbiol..

[37] Quan X.T., Siddiqi M.Z., Liu Q.Z., Lee S.M., Im W.T. (2020). *Devosia ginsengisoli* sp. nov., isolated from ginseng cultivation soil. Int. J. Syst. Evol. Microbiol..

[38] Weisburg W.G., Barns S.M., Pelletier D.A., Lane D.J. (1991). 16S ribosomal DNA amplification for phylogenetic study. J. Bacteriol..

[39] Yoon S.H., Ha S.M., Kwon S., Lim J., Kim Y. (2017). Introducing EzBioCloud: A taxonomically united database of 16S rRNA gene sequences and whole-genome assemblies. Int. J. Syst. Evol. Microbiol..

[40] Tamura K., Stecher G., Peterson D., Filipski A., Kumar S. (2013). MEGA6: Molecular evolutionary genetics analysis version 6.0. Mol. Biol. Evol..

[41] Saitou N., Nei M. (1987). The neighbor-joining method: A new method for reconstructing phylogenetic trees. Mol. Biol. Evol..

[42] Siddiqi M.Z., Im W.T. (2020). *Hankyongella ginsenosidimutans* gen. nov., sp. nov., isolated from mineral water with ginsenoside coverting activity. Antonie Van Leeuwenhoek.

[43] Akter S., Huq M.A. (2020). Biologically rapid synthesis of silver nanoparticles by *Sphingobium* sp. MAH-11 and their antibacterial activity and mechanisms investigation against drug-resistant pathogenic microbes. Artif. Cells Nanomed. Biotechnol..

[44] Govindaraju K., Kiruthiga V., Kumar G., Singaravelu G. (2009). Extracellular synthesis of silver nanoparticles by a marine alga, *Sargassum Wightii grevilli* and their antibacterial effects. J. Nanosci. Nanotechnol..

[45] Cavaliere E., De Cesari S., Landini G., Riccobono E., Pallecchi L., Rossolini G.M., Gavioli L. (2015). Highly bactericidal Ag nanoparticle films obtained by cluster beam deposition. Nanomed. Nanotechnol. Biol. Med..

[46] Kumar-Krishnan S., Prokhorov E., Hernández-Iturriaga M., Mota-Morales J.D., Vázquez-Lepe M., Kovalenko Y., Luna-Bárcenas G. (2015). Chitosan/silver nanocomposites: Synergistic antibacterial action of silver nanoparticles and silver ions. Eur. Polym. J..

[47] Sotiriou G.A., Pratsinis S.E. (2010). Antibacterial activity of nanosilver ions and particles. Environ. Sci. Technol..

[48] Sondi I., Salopek-Sondi B. (2004). Silver nanoparticles as antimicrobial agent: A case study on *E. coli* as a model for Gram-negative bacteria. J. Colloid Interface Sci..

[49] Yamanaka M., Hara K., Kudo J. (2005). Bactericidal actions of a silver ion solution on *Escherichia coli*, studied by energy-filtering transmission electron microscopy and proteomic analysis. Appl. Environ. Microbiol..

